# Growth performance of broiler chickens fed on feeds with varying mixing homogeneity

**DOI:** 10.1016/j.vas.2022.100263

**Published:** 2022-07-08

**Authors:** Alexandre Gomes Rocha, Paulo Dilkin, Roberto Montanhini Neto, César Schaefer, Carlos Augusto Mallmann

**Affiliations:** a Delacon Biotechnik GmbH, Langwiesen 24, 4209 Engerwitzdorf, Austria; b Federal University of Santa Maria, Department of Preventive Veterinary Medicine, Laboratory of Mycotoxicological Analysis, Santa Maria, Rio Grande do Sul, 97.105-900, Brazil; c Cooperativa Central Aurora Alimentos, Department of Animal Nutrition, Chapecó, Santa Catarina, 89.803-901, Brazil

**Keywords:** Feed homogeneity, Broilers, Body weight uniformity, Performance, Feed mixing

## Abstract

In feed manufacturing, the need for an effective combination of ingredients to produce a complete feed for animals is well known. However, limited quantitative data illustrate the effect of low homogeneity on the performance of animals. This study was conducted to determine the coefficient of variation (CV) of feed mixtures produced with different mixing times and the effects of these feeds with varying homogeneity consumed by 1–40-day-old broilers on their feed intake, body weight gain, feed conversion, mortality, and body weight uniformity. The mixing times used were 30, 60, 90, and 120 s, and the CV obtained from the feeds ranged from 49.5% to 5.4%. A linear decrease was observed in the feed CV with increasing mixing time, but sometimes, a longer mixing time did not present the lowest CV. The influence of the lack of feed homogeneity on the performance of broilers was mainly observed in body weight gain and, in some cases, in feed conversion. The effects on feed intake were more consistent for broilers older than 22 days, increasing body weight gain without affecting feed conversion. Our results suggest that feeds with CVs up to 22.6% had no adverse effects on the performance of broilers older than 12 days, and the lack of uniformity in broiler body weight was not influenced by feed homogeneity.

## Highlights

• We investigated the performance of broilers fed feeds of varying homogeneity.
• The coefficient of variation (CV) of feeds with different mixing times was studied.
• A linear decrease in feed CV with increasing mixing time was observed.
• The lack of uniformity in broiler weight was not influenced by feed homogeneity.

## 1 Introduction

Mixing ingredients is an essential process in feed manufacturing, as these ingredients must be combined effectively to be supplied as a complete feed to animals (McCoy, Behnke, Hancock & Mcellhiney, 1994). The mixing process should create a random distribution of ingredients in all mass portions to provide the animals with an adequate daily nutrient intake (Çiftci & Ercan, 2003). With the increased use of low-inclusion ingredients, such as industrial amino acids and other additives, efficient blending has become even more critical for an adequate nutrient supply (Groesbeck et al., 2007).

Moreover, feed homogeneity is desirable, if not necessary, to maximize nutrient use as, to improve growth, production, and health, each animal must receive a balanced feed that provides nutrients and additives at adequate concentrations (McCoy et al., 1994), especially when animals consume the feed with a low daily intake, such as weanling piglets and chicks (Ensminger, Oldfield & Heinemann, 1990).

The uniformity and mean body weight of a batch are essential parameters in broiler production (Dalanezi et al., 2005; Molenaar, Reijrink, Meijerhof & van den Brand, 2008), as is often emphasized in the meatpacking industry because the automation process requires uniform carcasses to achieve high-quality standards for processed products and to maximize the yield (Griffin, Renema, Robinson & Zuidhof, 2005). However, handling processes affect the uniformity of a production batch, and the homogeneity of slaughter body weights can be influenced by events during the production period (Judice, Muniz & Carvalheiro, 1999).

Despite the importance of feed nutrient homogeneity, few studies have correlated this with animal performance or focused on the level of feed homogeneity that would affect animal performance (Çiftci & Ercan, 2003). Moreover, no quantitative data on the effect of low homogeneity on animal performance has been published (Groesbeck et al., 2007; McCoy et al., 1994).

Behnke (1996) suggested that more research is warranted to elucidate the level of homogeneity required to improve animal performance, and formulation, nutrition, and regulatory affairs are still the basis of assumptions for the homogeneity of nutrients or additives with few scientific results supporting this assumption.

Considering this context, we aimed to determine the effects of feed with varying homogeneity consumed by 1–40-day-old broilers on feed intake, body weight gain, feed conversion, mortality, and body weight uniformity in this study.

## 2 Materials and methods

### 2.1 Animals

The experimental protocol was ethically and methodologically approved by the Animal Ethics Committee of the Federal University of Santa Maria, Santa Maria, RS, Brazil, under opinion number 066/2013.

A total of 2520 Cobb 500® one-day male broiler chicks were selected from the eggs of 49-week-old breeders. All chicks were weighed individually with an electronic scale Marte BL 3200H with precision of 0.01 g (Marte Científica & Instrumentação Industrial Ltda – Santa Rita do Sapucaí – MG - Brazil), but the numbers were rounded to the nearest integer; for instance, chicks weighing 42.5–43.5 g were considered having a mean body weight of 43.0 g.

The study included chicks weighing 43–51 g, with each replicate comprising five chicks of 43 g, eight of 44 g, eleven of 45 g, twelve of 46 g, eight of 47 g, nine of 48 g, seven of 49 g, seven of 50 g, and three of 51 g. Total of nine replicates per treatment with 70 chicks per replicate.

These chicks were housed in four randomly assigned treatments with different feed mixing times. They were housed in a conventional aviary with wire mesh sides and double curtains for insulation, with concrete-floored pens (2.0 *m* × 3.0 m) covered with a layer of wood shavings. Each pen was provided with a tubular feeder, a pendular drinking trough, and a heating brooder with electrical resistance.

The birds received water *ad libitum* from housing to loading for slaughter and were fed *ad libitum* from housing to 6 h before loading for slaughter. The drinking troughs were sanitized daily.

Minimum and maximum temperatures were recorded daily during the rearing period, and the minimum and maximum temperatures were 26 °C and 34 °C, 26 °C and 31 °C, 20 °C and 32 °C, 20 °C and 29 °C, 16 °C and 31 °C, and 18 °C and 29 °C on weeks 1–6, respectively.

The light program included 24 h of light on days 1–6, 1 h of darkness on day 7, 2 h of darkness on day 8, 3.5 h of darkness on day 9, 6 h of darkness on days 10–12, 8 h of darkness on days 13–21, and 6 h of darkness from day 22 to slaughter. The lighting was provided with white incandescent bulbs and the light intensity was not noted.

Dead birds were weighed, and the feed intake per pen was recorded for corrections at the end of each production phase.

The production phases were established as phases 1 (1–12 days), 2 (13–21 days), 3 (22–33 days), and 4 (34–40 days). At the end of each production phase, data on the body weight of each bird and the feed consumption of each pen were collected.

### 2.2 Feed treatments

Feeds were calculated according to Rostagno et al. (2011) recommendations for superior performance in male broilers (Table 1). The nutrient contents of the feeds are presented in Table 2.

**Table 1:** Ingredients of all feed phases used to feed broilers. Amounts are described in kilograms.

Ingredient	Phase 1	Phase 2	Phase 3	Phase 4
Corn	488.00	594.67	640.00	673.33
Soybean meal	224.84	108.85	72.90	41.91
Meat and bone meal	32.00	20.00	12.00	6.67
Full-fat soybean meal	230.67	254.67	254.67	260.00
Limestone	5.73	7.40	7.67	7.33
Salt	4.80	4.93	4.67	4.53
Vitamin mineral premix^1^	2.00	1.90	1.50	0.90
Antimicotoxin additive^2^	2.00	–	–	–
L-Lysine HCl (99%)^3^	2.15	1.91	1.67	1.65
DL-Methionine (99%)^4^	3.61	2.42	1.97	1.64
L-Threonine (98%)^5^	1.51	1.09	0.85	0.75
L-Valine (96,5%)^6^	1.19	0.74	0.57	0.45
Choline chloride (60%)	0.58	0.50	0.50	0.46
Protease enzyme^7^	0.13	0.13	0.13	0.13
Phytase enzime^8^	0.02	0.02	0.02	0.02
Xylanase + beta-glucanase^9^	0.05	0.05	0.05	0.05
Antioxidant additive^10^	0.13	0.13	0.13	0.13
Anticoccidial 1^11^	0.50	0.50	–	–
Anticoccidial 2^12^	–	–	0.55	–
Antibiotic growth promoter 1^13^	0.04	0.04	–	–
Antibiotic growth promoter 2^14^	–	–	0.10	–
Microtracer® F-Blue	0.05	0.05	0.05	0.05
Metabolizable energy (MJ/kg)	12.54	13.17	13.38	13.59

^1^In each kilogram: Vit. A (4275 KUI), vit. D_3_ (1395 KUI), vit. E (22,500 mg), vit. K_3_ (1350 mg), vit. B_1_ (1350 mg), vit. B_2_ (3600 mg), vit. B_6_ (1800 mg), vit. B_12_ (9900 μg), pantothenic acid (9000 mg), nicotinic acid (18,000 mg), folic acid (900 mg), biotin (76,500 μg), Cu (7200 mg), Fe (27,000 mg), Mn (27,000 mg), I (450 mg), Zn (45,000 mg) and Se (180 mg). ^2^Adsorb Afla - bentonite. ^3^AjiLys® 99. ^4^Rhodimet NP 99 (2-amino-4(methylthio) butanoic acid). ^5^L-Threonine 98%. ^6^L-Valine Feed Grade. ^7^Poultrygrow TM 250. ^8^Ronozyme HiPhos (M). ^9^Rovabio Excel AP. ^10^Feedguard. ^11^Maxiban 80 80. ^12^Coxistac Premix 12%. ^13^Stafac 500. ^14^Flavimpex 80.

**Table 2:** Nutrient contents in all the feed in phases 1, 2, 3, and 4. Amino acids are presented as totals.

Nutrient	Phase				Nutrient	Phase			
	1	2	3	4		1	2	3	4
Dry matter	89.78	89.78	89.75	89.70	M + C	1.11	0.90	0.82	0.76
Crude protein	24.57	20.00	18.21	16.93	Threonine	1.06	0.86	0.77	0.71
Crude fiber	3.12	2.86	2.77	2.72	Tryptophan	0.30	0.24	0.22	0.20
Ether extract	7.28	7.82	7.84	7.96	Valine	1.25	1.00	0.90	0.83
Ash	5.64	4.65	4.20	3.85	Arginine	1.73	1.40	1.27	1.18
Calcium	0.80	0.67	0.57	0.49	Histidine	0.64	0.54	0.50	0.47
Phosphorus	0.59	0.46	0.40	0.35	Isoleucine	1.04	0.84	0.76	0.71
Lysine	1.59	1.28	1.15	1.07	Leucine	2.02	1.74	1.63	1.56
Methionine	0.73	0.57	0.51	0.46	Phenylalanine	1.19	0.98	0.90	0.85
Cysteine	0.38	0.33	0.31	0.30	Sodium	0.22	0.22	0.21	0.20

M + C: methionine + cysteine. Phosphorus: Total phosphorus.

A mixing cycle was produced for each production phase with 1.5 tons of feed per treatment. The treatments were feed mixing times of 30, 60, 90, and 120 s. The mixing time was considered after the addition of pre-mixed micro-ingredients.

The used ingredients were corn, soybean meal, full-fat soybean, meat and bone meal, limestone, salt, anti-mycotoxin additive, vitamin and mineral premix, l-lysine HCl, DL-methionine, l-threonine, l-valine, choline chloride powder, phytase, xylanase, and protease enzymes, antioxidant additive, anticoccidial additives and growth promoters, and Microtracer® F-Blue (MFB). No liquid ingredients were added. The ingredients from the vitamin mineral premix to MFB were pre-mixed in a Y-mixer before addition to the mixer (U-type horizontal mixer, 1.5 m diameter and 3.6 m length, 35 rpm, Imoto® Motors and Machines Ltd., Brazil).

After the pre-established mixing time of each treatment, the mixer was turned off, and 10 feed samples were collected from inside the mixer. Samples 1 to 6 were collected from the surface of the mass with a plastic ladle, and samples 7 to 10 were collected from the middle to the base of the mixer using a plastic probe with detachable lid, attached to a metal rod. The samples were analyzed to determine the coefficient of variation (CV) of the mixture. The CVs of the entire period were weighed according to the quantities consumed in each phase.

Feeds were then unloaded into the reservoir below the mixer and transported through a screw conveyor (0.3 m in diameter and 1.0 m in length) into a packer. The first 50 kg portion of each treatment was discarded before packing the feeds for the experiment. Feeds were offered to the birds in mash form.

### 2.3 Statistical analysis

Statgraphics Centurion XV (Statgraphics Technologies Inc., Warrenton, VA, USA) was used for the statistical analyses.

Simple regression analysis with a single factor was performed on the feed mixture CVs and the zootechnical performance data. For the zootechnical data, each pen represented an experimental unit. The mean body weight gain, feed intake, feed conversion, and bird viability were calculated for each repetition of each production phase. Linear and quadratic effects were evaluated. The mixing time was considered an independent variable, and the feed mixing CV as a dependent variable. The feed mixing CV was considered an independent variable, and the data on animal performance was the dependent variable. The prediction equations are presented for significant models.

The data that presented a normal distribution were subjected to mean comparison tests, and the other data were subjected to median comparison tests.

The body weight uniformity of the broilers was verified by comparing the means of the body weight CVs from each pen. The uniformity of body weight distribution in each treatment considering all birds was also confirmed with the kurtosis and asymmetry indices of the distributions by comparing the medians with the nonparametric Kruskal-Wallis test.

## 3 Results

The analyzed nutrient compositions of the feed exhibited minor variations among treatments (Table 3); however, when weighed over the entire period, there were no evident variations. In treatments 1, 2, 3, and 4, total protein consumption was 873, 872, 873, and 863 g, respectively; total fat consumption was 350, 365, 351, and 352 g, respectively; and total Lysine consumption was 51, 52, 52, and 52 g per broiler, respectively.

**Table 3:** Analyzed nutrient contents in the feed in phases 1, 2, 3, and 4 according to treatments. Results are presented in percentage. Mean geometric diameter (MGD) of feed particles is expressed in micrometers. GSD: Geometric standard deviation of the particle size.

Nutrient	Treatments - Phase 1				Treatments - Phase 2				Treatments - Phase 3				Treatments - Phase 4			
	30 s	60 s	90 s	120 s	30 s	60 s	90 s	120 s	30 s	60 s	90 s	120 s	30 s	60 s	90 s	120 s
Dry matter	88.95	89.01	88.99	89.12	88.97	89.11	89.40	89.26	88.95	88.91	89.16	89.23	88.20	88.02	88.14	88.23
Crude protein	25.09	25.08	25.51	24.52	20.55	20.76	20.15	20.64	19.25	19.19	18.94	18.62	17.95	16.90	17.17	17.45
Crude fiber	2.22	2.61	2.67	2.84	1.39	1.85	1.99	2.07	1.50	1.91	1.94	1.91	1.65	1.83	1.81	2.07
Ether extract	5.61	5.51	6.35	7.86	8.36	8.44	7.95	8.28	7.83	8.78	8.05	7.45	8.52	7.76	7.98	8.29
Ash	5.64	5.51	6.35	7.86	4.58	4.40	4.58	4.50	3.94	4.24	3.98	4.21	3.88	3.86	3.74	3.70
Calcium	0.71	0.89	0.80	0.78	0.75	0.65	0.69	0.65	0.45	0.47	0.55	0.58	0.47	0.46	0.49	0.47
Total phosphorus	0.57	0.61	0.60	0.57	0.44	0.35	0.42	0.47	0.37	0.35	0.37	0.38	0.33	0.39	0.35	0.35
Lysine	1.50	1.64	1.63	1.52	1.33	1.24	1.23	1.29	1.07	1.10	1.11	1.09	0.99	0.99	1.00	1.02
Methionine	0.55	0.68	0.62	0.65	0.65	0.49	0.50	0.44	0.38	0.40	0.42	0.42	0.38	0.41	0.41	0.39
Cysteine	0.36	0.38	0.37	0.35	0.31	0.33	0.33	0.31	0.28	0.32	0.32	0.31	0.30	0.28	0.29	0.31
Met + Cys	0.91	1.06	1.00	1.00	0.95	0.82	0.83	0.75	0.65	0.72	0.74	0.73	0.67	0.69	0.70	0.70
Threonine	1.02	1.14	1.11	1.11	0.98	0.89	0.89	0.84	0.78	0.77	0.81	0.79	0.71	0.70	0.72	0.73
Valine	1.14	1.23	1.26	1.17	1.03	0.95	0.96	0.93	0.81	0.88	0.85	0.86	0.79	0.77	0.80	0.82
Alanine	1.12	1.18	1.25	1.11	1.03	1.02	1.01	0.96	0.89	0.92	0.90	0.94	0.87	0.85	0.89	0.89
Arginine	1.47	1.63	1.64	1.62	1.36	1.35	1.35	1.40	1.06	1.04	1.17	1.21	1.06	0.99	0.99	1.06
Glycine	1.14	1.18	1.24	1.18	0.93	0.88	0.90	0.93	0.75	0.82	0.78	0.81	0.73	0.68	0.71	0.72
Histidine	0.54	0.63	0.63	0.61	0.53	0.53	0.51	0.53	0.49	0.52	0.53	0.50	0.47	0.46	0.43	0.46
Isoleucine	0.95	1.03	1.06	0.97	0.83	0.79	0.79	0.80	0.69	0.73	0.73	0.71	0.65	0.64	0.66	0.67
Leucine	1.86	1.97	2.05	1.97	1.71	1.65	1.65	1.68	1.49	1.57	1.57	1.54	1.47	1.40	1.47	1.50
Phenylalanine	1.14	1.22	1.28	1.22	1.06	0.99	0.98	0.99	0.86	0.93	0.90	0.91	0.85	0.86	0.85	0.88
Serine	1.12	1.22	1.22	1.20	0.99	0.97	0.96	0.93	0.86	0.89	0.90	0.87	0.81	0.79	0.81	0.82
Tyrosine	0.78	0.85	0.86	0.84	0.74	0.69	0.68	0.69	0.61	0.61	0.65	0.66	0.60	0.59	0.57	0.61
MGD (μm)	995	961	996	1.045	1.122	1.113	1.103	1.092	1.117	1.113	1.073	1.107	1.087	1.078	1.132	1.115
GSD	2.07	2.02	2.02	1.97	2.03	1.98	2.01	1.99	2.01	2.06	2.04	2.08	2.05	2.14	2.05	2.04

In this study, the homogeneity of the ingredients and the nutrients was indirectly measured using the CVs obtained with the MFB. The CVs were 35.9% – 7.5% (Table 4), 49.5% – 5.4% (Table 5), 34.8% – 5.4% (Table 6) and 40.8% – 5.8% (Table 7) in phases 1, 2, 3 and 4, respectively, and 39.5% – 5.7% (Table 8) in the total period.

**Table 4:** Effect of mixing time on the coefficient of variation (CV) of the feeds, and effect of feed CV on the performance of broilers in phase 1 (1–12 days of age).

Treatment	CV^1^ (%)	FI^2^ (g)	BWG^3^ (g)	FCR^4^	Viab.^5^ (%)
30 s	35.9	400.56	366.62	1.093^BC^	96.35
60 s	20.4	400.78	357.25	1.122^A^	98.57
90 s	10.7	401.67	364.00	1.104^AB^	98.09
120 s	7.5	402.33	372.94	1.079^C^	97.94
CV^6^	NA^7^	1.53	2.37	2.072	2.85
P - Linear	<0.0001	0.5396	0.2997	0.4812	0.1834
r	−0.9614	−0.1057	−0.1777	0.1212	−0.2269
R^2^	92.4263	1.1167	3.1595	1.4698	5.1462
SE	3.1240	6.1866	8.6301	0.0229	2.7490
P - Quadratic	<0.0001	0.5785	0.6764	0.9679	0.1301
r	−0.8979	−0.0958	−0.0720	0.0069	−0.2571
R^2^	80.6256	0.9170	0.5187	0.0048	6.6099
SE	4.9965	6.1928	8.7469	0.0231	2.7278

^1^CV (%): coefficient of variation of feed. ^2^FI: feed intake. ^3^BWG: body weight gain. ^4^FCR: feed conversion ratio. ^5^Viab.: viability. ^6^CV: coefficient of variation in zootechnical data. ^7^NA: not applicable. r: correlation coefficient. R^2^: Coefficient of determination. SE: standard error of the estimate. P: probability of significance of regression analysis. Different uppercase letters in the column indicate differences according to the Kruskal-Wallis median comparison test (*p <* 0.05).

CVphase1=42.335−0.3162×time,.

$\text{CV}\;\text{phase}\;1\;=\;31.702\;-\;0.00193807\;\times \;\text{tim}e^{2}$.

where.

CV phase 1 = coefficient of variation of the feed used for phase 1 (%) and

Time = mixing time (s).

**Table 5:** Effect of the mixing time on the coefficient of variation (CV) of the feeds, and effect of the feed CV on the performance of broilers in phase 2 (13–21 days of age).

Treatment	CV^1^ (%)	FI^2^ (g)	BWG^3^ (g)	FCR^4^	Viab.^5^ (%)
30 s	49.5	960.67	697.37	1.378	99.84
60 s	22.6	964.33	703.82	1.370	100.00
90 s	8.9	969.56	705.70	1.374	99.84
120 s	5.4	957.89	696.16	1.376	99.83
CV^6^	NA^7^	1.85	2.45	1.150	0.41
P - Linear	<0.0001	0.7606	0.6944	0.7222	0.9599
r	−0.9414	−0.0526	−0.0678	0.0614	0.0087
R^2^	88.6268	0.2767	0.4569	0.3765	0.0075
SE	6.0137	18.0529	17.3776	0.0160	0.4163
P - Quadratic	<0.0001	0.6766	0.5740	0.5790	0.8921
r	−0.8674	−0.0720	−0.0969	0.0956	−0.0234
R^2^	75.2411	0.5178	0.9388	0.9147	0.0549
SE	8.8729	18.0310	17.3357	0.0160	0.4162

^1^CV (%): coefficient of variation of feed. ^2^FI: feed intake. ^3^BWG: body weight gain. ^4^FCR: feed conversion ratio. ^5^Viab.: viability. ^6^CV: coefficient of variation in zootechnical data. ^7^NA: not applicable. r: correlation coefficient. R^2^: Coefficient of determination. SE: standard error of the estimate. P: probability of significance of regression analysis.

CVphase2=58.09−0.4864×time,.

$\text{CV}\;\text{phase}\;2\;=\;41.4623\;-\;0.00294109\;\times \;\text{tim}e^{2}$,.

where CV phase 2 = coefficient of variation of the feed used for phase 2 (%) and

Time = mixing time (s).

**Table 6:** Effect of the mixing time on the coefficient of variation (CV) of the feeds, and effect of the feed CV on the performance of broilers in phase 3 (22–33 days of age).

Treatment	CV^1^ (%)	FI^2^ (g)	BWG^3^ (g)	FCR^4^	Viab.^5^ (%)
30 s	34.8	1988.44^b^	1221.43^b^	1.629	98.55
60 s	11.9	2017.78^ab^	1251.57^a^	1.612	99.19
90 s	9.8	2031.33^a^	1256.92^a^	1.616	99.35
120 s	5.4	2027.89^a^	1254.08^a^	1.617	99.35
CV^6^	NA^7^	1.71	2.28	1.199	1.18
P - Linear	<0.0001	0.0035	0.0016	0.0713	0.0969
r	−0.7672	−0.4744	−0.5066	0.3042	−0.2810
R^2^	58.8592	22.5092	25.6621	9.2523	7.8938
SE	6.2509	30.8575	24.8898	0.0188	1.1419
P - Quadratic	<0.0001	0.0035	0.0016	0.0712	0.0970
r	−0.6503	−0.4739	−0.5062	0.3043	−0.2809
R^2^	42.2890	22.4542	25.6191	9.2575	7.8925
SE	7.4035	30.8685	24.8970	0.0188	1.1420

^1^CV (%): coefficient of variation of feed. ^2^FI: feed intake. ^3^BWG: body weight gain. ^4^FCR: feed conversion ratio. ^5^Viab.: viability. ^6^CV: coefficient of variation in zootechnical data. ^7^NA: not applicable. r: correlation coefficient. R^2^: Coefficient of determination. SE: standard error of the estimate. P: probability of significance of regression analysis. Different lowercase letters in the column indicate differences by the Student-Newman-Keuls mean comparison test (*p <* 0.05).

CVphase3=34.33−0.216633×time.

$\text{CV}\;\text{phase}\;3\;=\;26.2165\;-\;0.00120504\;\times \;\text{tim}e^{2}$.

FIphase3=2,047.22−1,70,651×CVphase3.

$\text{FI}\;\text{phase}\;3\;=\;2,031.45\;-\;0.036204\;\times \;\text{CV}\;\text{phase}\;3^{2}$.

BWGphase3=1,273.13−1.50057×CVphase3.

$\text{BWG}\;\text{phase}\;3\;=\;1,259.27\;-\;0.0318471\;\times \;\text{CV}\;\text{phase}\;3^{2}$.

where CV phase 3 = coefficient of variation of the feed used for phase 3 (%).

Time = mixing time (s).

FI = feed intake in phase 3 (g) and

BWG = body weight gain in phase 3 (g).

**Table 7:** Effect of the mixing time on the coefficient of variation (CV) of the feeds, and effect of the feed CV on the performance of broilers in phase 4 (34–40 days of age).

Treatment	CV^1^ (%)	FI^2^ (g)	BWG^3^ (g)	FCR^4^	Viab.^5^ (%)
30 s	40.8	1073.67	573.29	1.876	99.19
60 s	10.0	1088.67	576.80	1.890	98.70
90 s	10.8	1106.89	571.69	1.939	98.84
120 s	5.8	1111.89	578.02	1.926	99.83
CV^6^	NA^7^	3,56	5.24	4.229	1.15
P - Linear	<0.0001	0.0438	0.8166	0.1734	0.8722
r	−0.8345	−0.3379	−0.0041	−0.2320	−0.0278
R^2^	69.6357	11.4178	−0.1605	5.3816	0.0772
SE	7.9167	37.2812	30.5629	0.0796	1.1532
P - Quadratic	−0.0001	0.0501	0.8393	0.1752	0.9702
r	−0.7399	−0.3290	−0.0350	−0.2310	0.0064
R^2^	54.741	10.8264	0.1226	5.3363	0.0042
SE	9.6652	37.4054	30.5687	0.0797	1.1536

^1^CV (%): coefficient of variation of feed. ^2^FI: feed intake. ^3^BWG: body weight gain. ^4^FCR: feed conversion ratio. ^5^Viab.: viability. ^6^CV: coefficient of variation in zootechnical data. ^7^NA: not applicable. r: correlation coefficient. R^2^: coefficient of determination. SE: standard error of the estimate. P: probability of significance of regression analysis.

CVphase4=42.91−0.347367×time.

$\text{CV}\;\text{phase}\;4\;=\;30.5002\;-\;0.00202115\;\times \;\text{tim}e^{2}$.

FIphase4=1,110.98−0.931639×CVphase4.

$\text{FI}\;\text{phase}\;4\;=\;1,104.12\;-\;0.0184613\;\times \;\text{CV}\;\text{phase}\;4^{2}$.

where

CV phase 4 = coefficient of variation of the feed used for phase 4 (%).

Time = mixing time (s) and FI = feed intake in phase 4 (g).

**Table 8:** Effect of mixing time on the coefficient of variation (CV) of the feeds, and effect of feed CV on the performance of broilers in the entire production period (1–40 days of age).

Treatment	CV^1^ (%)	FI^2^ (g)	BWG^3^ (g)	FCR^4^	Viab.^5^ (%)
30 s	39.5	4423.33	2858.71	1.547	93.97
60 s	14.5	4471.56	2889.44	1.547	96.51
90 s	9.9	4509.44	2898.40	1.556	96.19
120 s	5.7	4500.00	2901.37	1.551	96.98
CV^6^	NA^7^	1.80	1.81	0.858	3.21
P - Linear	<0.0001	0.0156	0.0528	0.3941	0.0250
r	−0.8587	−0.4003	−0.3254	−0.1464	−0.3731
R^2^	73.7404	16.0228	10.5908	2.1441	13.9227
SE	6.2659	74.8118	50.1754	0.0134	2.8949
P - Quadratic	<0.0001	0.0176	0.0556	0.4170	0.0253
r	−0.7618	−0.3934	−0.3218	−0.1395	−0.3724
R^2^	58.0285	15.4731	10.3554	1.9472	13.8659
SE	7.9217	75.0562	50.2435	0.0134	2.8958

^1^CV (%): coefficient of variation of feed. ^2^FI: feed intake. ^3^BWG: body weight gain. ^4^FCR: feed conversion ratio. ^5^Viab.: viability. ^6^CV: coefficient of variation in zootechnical data. ^7^NA: not applicable. r: correlation coefficient. R^2^: coefficient of determination. SE: standard error of the estimate. P: probability of significance of regression analysis.

CVphaseT=41.84−0.304233×time.

$\text{CV}\;\text{phase}\;T\;=\;30.9774\;-\;0.0017711\;\times \;\text{tim}e^{2}$.

BWGphaseT=2,913.85−1.41234×CVphaseT.

$\text{BWG}\;\text{phase}\;T\;=\;2,900.75\;-\;0.0273634\;\times \;\text{CV}\;\text{phase}\;T^{2}$.

FIphaseT=4,526.92−2.6725×CVphaseT.

$\text{FI}\;\text{phase}\;T\;=\;4,501.97\;-\;0.0514574\;\times \;\text{CV}\;\text{phase}\;T^{2}$.

Viab.phaseT=97.7237−0.0952147×CVphaseT.

$\text{Viab}.\;\text{phase}\;T\;=\;96.8491\;-\;0.00186178\;\times \;\text{CV}\;\text{phase}\;T^{2}$.

where

CV phase *T* = weighted coefficient of variation of all feeds (%).

Time = mixing time (s).

BWG phase *T* = body weight gain in the entire production phase (g).

FI phase *T* = feed intake in the entire production phase (g) and Viab.

phase *T* = viability in the entire production phase (%).

Each pen's mean zootechnical performance data showed a normal distribution according to the Shapiro-Wilk test (*p >* 0.05), except for feed conversion in phases 1 and 4, body weight gain in phase 4, and viability in all phases except phase 1. Body weight CVs at 12 days were adjusted to normality using a logarithmic transformation, and body weight CVs at 21, 33, and 40 days were normally distributed, according to the Shapiro-Wilk test (*p >* 0.05). Treatment did not affect the performance (linearly and quadratically) of broilers in phase 1 for body weight gain (*p >* 0.29 and *p* > 0.67), feed intake (*p >* 0.53 and *p* > 0.57), feed conversion (*p >* 0.48 and *p* > 0.96), and viability (*p >* 0.18 and *p* > 0.13) (Table 4). In phase 2, no differences (linearly and quadratically) were found in body weight gain (*p >* 0.69 and *p* > 0.57), feed intake (*p >* 0.76 and *p* > 0.67), feed conversion (*p >* 0.72 and *p* > 0.57), and viability (*p >* 0.95 and *p* > 0.89), but the mixing CV showed a significant linear correlation (*p <* 0.0001) with mixing time (Table 5). Linear and quadratic effects were observed for body weight gain (*p <* 0.002) and feed intake (*p <* 0.004) in phase 3 (Table 6). A linear effect was observed for feed intake (*p <* 0.05) in phase 4, with CVs of 40.8%, 10.0%, 10.8%, and 5.8%, corresponding to mixing times of 30, 60, 90, and 120 s, respectively (Table 7). There were trends of linear and quadratic effects for feed conversion (*p* < 0.08) and viability (*p* < 0.10) in phase 3. Finally, linear and quadratic effects were observed in the total period (1–40 days) for feed intake (*p <* 0.02) and viability (*p <* 0.03), and there was a trend of linear effect for body weight gain (*p* < 0.06), with weighted CVs of 39.5%, 14.5%, 9.9%, and 5.7%, corresponding to mixing times of 30, 60, 90, and 120 s, respectively (Table 8).

## 4 Discussion

Clark (2006) used a double ribbon Sprout Waldron mixer with mixing times of 10, 20, 30, 40, and 120 s to evaluate the homogeneity of only one ingredient, obtaining the following significant CV reductions at all production stages with increased mixing times: 144.3% to 3.4% in phase 1 (0–16 days), 129.4% to 3.5% in phase 2 (17–32 days), and 134.8% to 5.6% in phase 3 (33–41 days).

The amplitudes of the CVs herein observed were lower than those in the study by Clark (2006); moreover, linear and quadratic effects (*p <* 0.0001) of negative correlation were herein observed between mixing time and CV in all phases, and a linear effect with the highest coefficient of determination was also observed in all phases (Table 1, Table 2, Table 3, Table 4, Table 5).

McCoy et al. (1994) evaluated the lack of homogeneity represented by the indicator CVs in broiler feeds in two experiments: the first with one phase up to 21 days and the second with two phases up to 28 days of broiler age. The authors worked with feeds to meet 100% and 80% of nutritional requirements according to the National Research Council (1984) in experiments 1 and 2, respectively. A horizontal double ribbon mixer (53 rpm, 142-L capacity) was used to produce 80 kg of feed at each mixing cycle. In the first experiment, these authors worked with three treatments, represented by mixing times of 23, 57, and 91 s, obtaining CVs of 43.0%, 10.8%, and 13.1%, respectively, for NaCl; 50.0%, 14.8%, and 17.1%, respectively, for Microtracer® F-Red (MFR); and 47.6%, 12.0%, and 14.6%, respectively, for MFB. In the second experiment, with mixing times of 6, 20, and 91 s, the CVs were 40.5%, 12.1%, and 9.7%, respectively, for NaCl; 53.4%, 16.6%, and 11.3%, respectively, for MFR; and 53.9%, 17.0%, and 10.6%, respectively, for MFB.

In their first experiment, McCoy et al. (1994) reported no significant differences in daily body weight gain, daily feed intake, bone strength, bone mineralization, crude protein, carcass fat, and minerals between treatments (*p >* 0.12). Feed efficiency also showed no difference among treatments (*p >* 0.09), and according to the authors, CVs >10%–15% were adequate for maximum growth, lean meat production, bone development, and even for feed efficiency. In the second experiment, they obtained a quadratic effect for daily body weight gain (*p <* 0.04), feed intake (*p <* 0.10), and feed efficiency (*p <* 0.07) only when comparing the treatments corresponding to the 6 and 20 s mixing times. No difference was observed between mixing times of 20 s and 91 s.

In this study, no effects of treatments were observed on the performance of broilers in phase 1 (Table 4), despite this being the phase in which the lack of feed homogeneity might produce the greatest effects as the broilers consume only a few grams that must include all the essential nutrients (Ensminger et al., 1990). Clark (2006) showed a linear effect on body weight gain (*p <* 0.001), a quadratic effect on feed conversion (*p <* 0.001), and no effect on feed intake (*p >* 0.17) in phase 1 (1–16 days).

McCoy et al. (1994) showed that the effect observed in experiment 2 resulted from the deficiency of essential nutrients within feeds that met 80% of broiler requirements; thus, a lack of homogeneity would affect animal performance.

Clark (2006) observed a quadratic effect (*p <* 0.003) in phase 2 (17–32 days), with higher feed intake with CVs of 52.2%, 24.8%, and 17.1% under mixing times of 20, 30, and 40 s, respectively, compared to the CVs of 129.4% and 3.5% under 10 and 120 s, respectively. In the study herein, linear and quadratic effects (Table 6) were observed for body weight gain (*p <* 0.002) and feed intake (*p <* 0.004), and trends of linear and quadratic effects for feed conversion (*p* < 0.08) and viability (*p* < 0.10) in phase 3 (22–33 days).

Clark (2006) also reported no effect on performance in phase 3 (33–41 days), with CVs of 134.8%, 21.2%, 23.2%, 20.6%, and 5.6% for mixing times of 10, 20, 30, 40, and 120 s, respectively. In the entire period (1–41 days), the author observed a quadratic effect for body weight gain (*p <* 0.001) and feed intake (*p <* 0.002). In the study herein in the entire period (1–40 days) a trend of difference (*p <* 0.06) for body weight gain and a difference in feed intake (*p <* 0.02) were observed (Table 8).

Çiftci and Ercan (2003) studied broilers in three stages (1–14, 15–35, and 36–42 days) reared with three treatments represented by mixing times of 12, 35, and 225 s, and obtained chlorine ion CVs of 38.4%, 11.9%, and 7.4%, respectively, in phase 1; 30.4%, 11.3%, and 9.8%, respectively, in phase 2; and 21.5%, 14.6%, and 8.9%, respectively, in phase 3. There were no statistically significant differences in body weight gain, feed intake, feed conversion, mortality, carcass yield, abdominal fat, and CV of individual body weight among treatments. These authors concluded that the lack of uniformity did not affect the performance of the broilers, as the feeds were calculated to meet or exceed nutritional requirements according to the National Research Council (1984) and that the broilers could tolerate CVs of up to 38.4%.

**Fig. 1:**
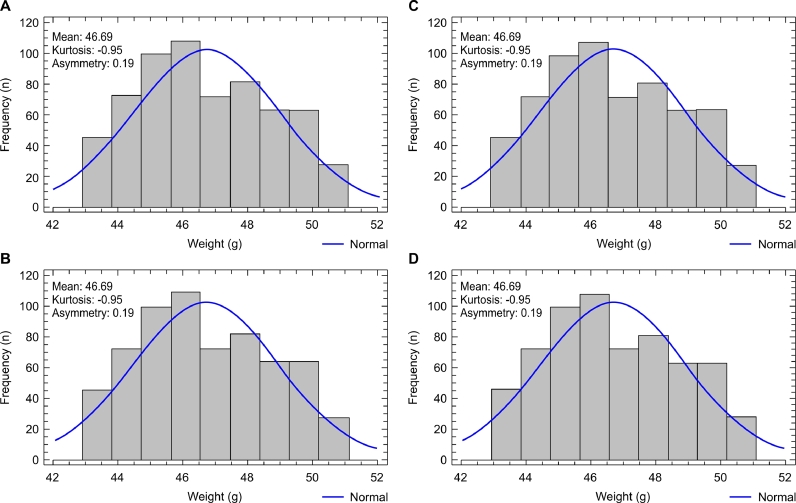
Graph of frequency distribution of body weights of 1-day-old male broilers. (A) 30 s feed mixing treatment. (B) 60 s feed mixing treatment. (C) 90 s feed mixing treatment. (D) 120 s feed mixing treatment.

The analysis of body weight homogeneity of the broilers in the present study showed that on day 1, the CV for body weight, kurtosis, and asymmetry were equal among treatments, highlighting the negative kurtosis and moderate positive asymmetry values (Table 9, Fig. 1). The analysis of body weight dispersion measures within the other phases showed a significant difference only for kurtosis in phase 3, with the most homogeneous distribution in the treatments with CVs of 11.9% and 9.8% (mixing times of 60 and 90 s, respectively).

**Table 9:** Mean body weights, mean coefficients of variation (weight CV), and mean kurtosis and asymmetry values of birds on days 1, 12, 22, 33, and 40, according to the treatments applied on day 1 and to the coefficients of variation of the feeds on the other days.

	Treatment - mixing time - day 1				
Parameter	30 s	60 s	90 s	120 s	*p
Body weight	46.69	46.69	46.69	46.69	1.000
Weight CV	4.82^C^	4.83^B^	4.83^B^	4.82^B^	0.579
Kurtosis	−0.95^C^	−0.95^B^	−0.95^B^	−0.95^B^	0.560
Asymmetry	0.19^C^	0.19^B^	0.19^B^	0.19^B^	0.560
	Treatment - Feed CV - Day 12 - Phase 1				
Parameter	38.9%	20.4%	10.7%	7.5%	*p
Body weight	413.57^b^	403.99^c^	410.72^b^	419.67^a^	<0.001
Weight CV	8.38^B^	9.10^A^	9.21^A^	8.59^A^	0.638
Kurtosis	1.54^B^	2.82^A^	3.15^A^	1.17^A^	0.642
Asymmetry	−0.69^B^	−1.05^A^	−1.06^A^	−0.81^A^	0.486
	Treatment - Feed CV - Day 21 - Phase 2				
Parameter	49.5%	22.6%	8.9%	5.4%	*p
Body weight	1110.70	1107.78	1116.30	1115.88	0.287
Weight CV	9.58^AB^	9.19^A^	9.10^A^	9.05^A^	0.858
Kurtosis	3.31^A^	3.02^A^	3.02^A^	2.68^A^	0.993
Asymmetry	−0.89^AB^	−1.12^A^	−1.03^A^	−1.07^A^	0.692
	Treatment - Feed CV - Day 33 - Phase 3				
Parameter	34.8%	11.9%	9.8%	5.4%	*p
Body weight	2332.04^b^	2359.38^a^	2372.95^a^	2369.93^a^	0.007
Weight CV	10.02^A^	9.58^A^	9.30^A^	8.59^A^	0.249
Kurtosis	3.00^abAB^	6.11^aA^	3.60^aA^	1.13^bA^	0.027
Asymmetry	−1.17^A^	−1.66^A^	−1.16^A^	−0.77^A^	0.087
	Treatment - Feed CV - Day 40 - Phase 4				
Parameter	40.8%	10.0%	10.8%	5.8%	*p
Body weight	2905.04^b^	2936.34^a^	2944.59^a^	2948.07^a^	0.042
Weight CV	10.05^AB^	9.24^A^	9.58^A^	9.15^A^	0.585
Kurtosis	4.83^A^	3.50^A^	4.94^A^	3.04^A^	0.433
Asymmetry	−1.56^A^	−1.28^A^	−1.39^A^	−1.14^A^	0.494
	**P values - within the treatment - between phases				
Weight CV	<0.0001	0.0002	0.0001	0.0002	
Kurtosis	<0.0001	<0.0001	0.0002	0.0002	
Asymmetry	<0.0001	0.0002	0.0002	0.0001	

Different lowercase letters in the row indicate significant differences (*p < 0.05) between treatments and within phases using the Student-Newman-Keuls test for weight CV, and using the Kruskal-Wallis test for mean weight, kurtosis, and asymmetry. Different uppercase letters in the column indicate a significant difference (**p < 0.05) within the treatment and between the phases for weight CV, kurtosis, and asymmetry according to the Kruskal-Wallis test.

The dispersion data in mixing times of 60, 90, and 120 s exhibited the same behavior (Table 9). Body weight, kurtosis, and asymmetry CVs were higher on days 12, 21, 33, and 40 compared to the broiler housing day, indicating that body weight dispersion had increased on day 12 and remained constant until broiler slaughter. Feed CVs between 5.4% and 22.6% were obtained in these treatments and phases.

Negative kurtosis in all treatments increased on day 12, becoming positive, and remained constant until the last weighing (Table 9). This demonstrates an excellent body weight dispersion, moving from a leptokurtic curve to a platykurtic curve.

**Fig. 2:**
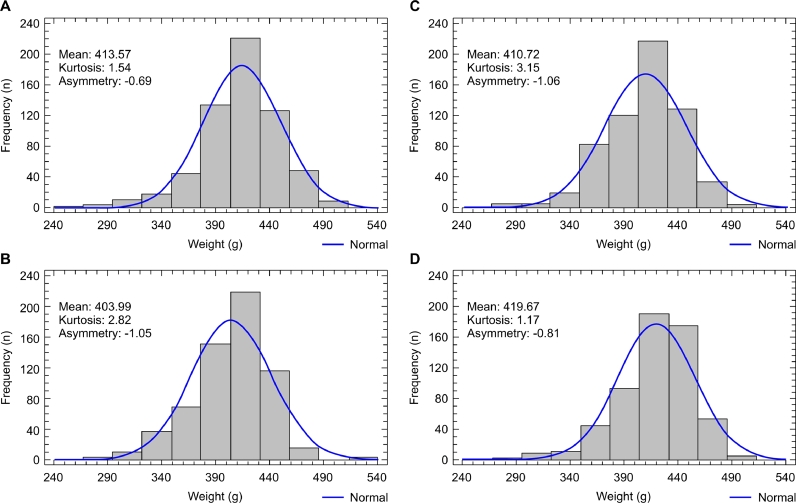
Graph of frequency distribution of body weights of 12-day-old male broilers. (A) 30 s feed mixing treatment: CV of 35.9%. (B) 60 s feed mixing treatment: CV of 20.4%. (C) 90 s feed mixing treatment: CV of 10.7%. (D) 120 s feed mixing treatment: CV of 7.5%.

**Fig. 3:**
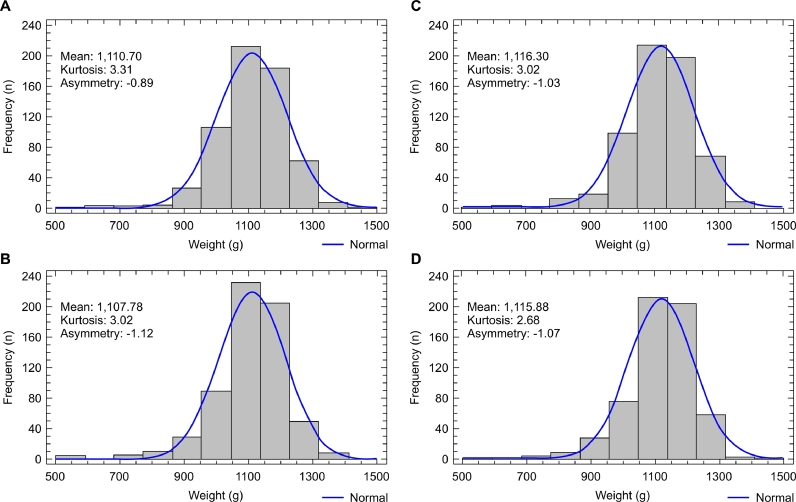
Graph of frequency distribution of the body weights of 21-day-old male broilers (A) 30 s feed mixing treatment: CV of 49.5%. (B) 60 s feed mixing treatment: CV of 22.6%. (C) 90 s feed mixing treatment: CV of 8.9%. (D) 120 s feed mixing treatment: CV of 5.4%.

**Fig. 4:**
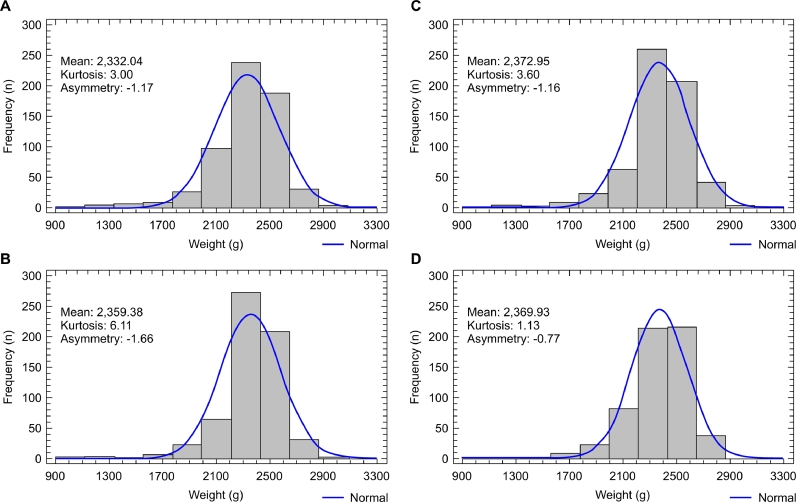
Graph of frequency distribution of body weights of 33-day-old male broilers (A) 30 s feed mixing treatment: CV of 34.8%. (B) 60 s feed mixing treatment: CV of 11.9%. (C) 90 s feed mixing treatment: CV of 9.8%. (D) 120 s feed mixing treatment: CV of 5.4%.

**Fig. 5:**
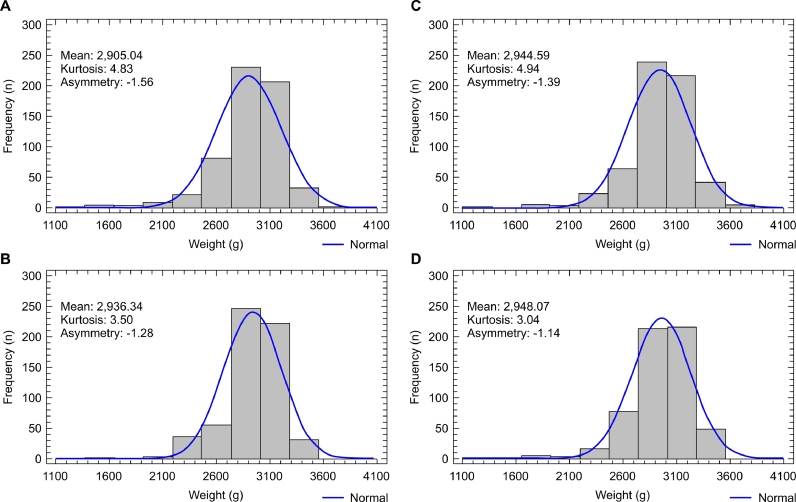
Graph of frequency distribution of body weights of 40-day-old male broilers. (A) 30 s feed mixing treatment: CV of 40.8%. (B) 60 s feed mixing treatment: CV of 10.0%. (C) 90 s feed mixing treatment: CV of 10.8%. (D) 120 s feed mixing treatment: CV of 5.8%.

Data asymmetry showed significant differences on day 12 compared to housing day in all treatments (Table 9). Moderate positive asymmetries in the housing day became moderately negative on days 12 and 21 (Fig. 2, Fig. 3) and strongly negative on days 33 and 40 (Fig. 4, Fig. 5), except for the mixing time of 120 s in phase 3, indicating progressive uniformity loss regardless of the feed CV.

Experiments that assess composition or nutritional requirements are typically based on the assumption that feeds are homogeneously mixed and randomly distributed and that all animals have the same feeding behavior. Therefore, the effects of these variables are often overlooked in experiments in which stalls or pens are the experimental units (Pritchard & Stateler, 1997). As no expression of choice or selection regarding the feed offered is considered to occur in animal experiments, each serving consumed would be uniform, definable, and as similar as possible to a nutritionally complete feed. It is acceptable that the integrity of the homogeneity achieved or the form of feed intake ultimately influences the nutritional status and animal performance (Coppock, Bath & Harris, 1981).

Cromwell et al. (2003) analyzed feeds produced in 25 experimental stations. They concluded that some of these could not have efficient mixtures, which shows that the variation in animal performance among treatments could be due to mixing errors.

However, Pritchard and Stateler (1997) reported that it was impossible to find specific data on the homogeneity of feeds in feeders as the feed is either considered equally consumed or rejected by all animals in an experimental unit.

Previous studies have indicated that the lack of variability in animal performance when non-homogenous feed is used can be explained based on the size of the sample, which can be the sample analyzed to detect the contents of indicators or the samples consumed by the animals daily. According to Ensminger et al. (1990), to ensure that each animal receives the correct amount of nutrients every day or week, the sample for analysis of the mixture should equal the amount consumed daily or weekly by the animal, respectively. This means that the servings ingested may vary, but the mean intake during the day or week would meet the total nutrient requirements.

McCoy et al. (1994) used feeds that met nutritional requirements and obtained the maximum performance of the broilers with CVs of 12% – 23%. In contrast, the industry recommendation is a CV of 10% or lower. McCoy et al. (1994) concluded that a CV of 20% represents the appropriate level of feed homogeneity for maximum broiler performance.

Çiftci and Ercan (2003) recommended using processes that guarantee a CV of up to 10% under non-experimental conditions when using feeds calculated to meet the minimum requirements of the birds.

The CV necessary for optimal performance depends highly on species, production stage, age of the animals, the indicator selected for the mixing homogeneity test, and the collection method of feed samples (Paulk, 2011). Regulatory matters must also be considered, as any feed marketed or distributed will deemed adulterated if a representative sample does not meet the label warranty statements (Traylor, Behnke, Stark, Hines & Hancock, 1994).

The present study showed linearly decreased CVs for feeds mixed from 30 to 120 s, but sometimes, a longer mixing time did not result in CV reduction. Remarkably, when lack of feed homogeneity affected the performance of broilers, it was observed in body weight gain and, sometimes, feed conversion. The effects on feed intake were more consistent for broilers aged over 22 days, promoting more significant body weight gain but with no difference in feed conversion. The birds tolerated the CVs above the standard recommended by regulatory agencies for feeds meeting nutritional requirements, with no effect on performance and body weight uniformity. The non-uniformity of broiler body weight did not depend on feed homogeneity up to the maximum feed CV observed in this study. Such body weight dispersion in broiler production likely depends on other factors not analyzed in this study. The lack of effect with high CV feed can be explained by the number of daily meals, which would increase the probability of intake with adequate amounts of nutrients during the day or even in two-day periods.

The zootechnical results showed that the acceptable feed CVs were contrary to some regulatory recommendations. However, most of the feed CV evaluations are performed using samples collected from mixers, not considering that feed homogeneity may be altered until ingestion by the animals.

## 5 Conclusions

The findings of this study suggest that feeds with CVs up to 22.6% have no adverse effect on the performance of male broiler chicks aged over 12 days provided they are fed diets with adequate nutrient content. Feeds with a CV of 10.7% negatively influenced the performance of broilers aged up to 12 days; therefore, using feeds with a CV of up to 7.5% is recommended. Feed mixed for 30 s, with averaged CV of 39.5%, caused an adverse effect on feed intake and body weight gain for male broilers chickens from 1 to 40 days.

The lack of feed homogeneity, with CVs up to 49.5%, did not influence the homogeneity of broiler body weights during growth or slaughter.

To better understand the interactions of the forms of feeds (*e.g.*, mash or pelleted), loss of homogeneity after mixing, and CVs, further studies are warranted to evaluate different physical forms of feed and production processes and feed CVs, from production until consumption.

## Funding

This research received no specific grant from funding agencies in the public, commercial, or not-for-profit sectors.

## Declarations of Competing Interest

None
